# Nitrogen reduction combined with ET_c_ irrigation maintained summer maize yield and increased water and nitrogen use efficiency

**DOI:** 10.3389/fpls.2023.1180734

**Published:** 2023-06-22

**Authors:** Limin Gu, Xinyuan Mu, Jianshuang Qi, Baojun Tang, Wenchao Zhen, Laikun Xia

**Affiliations:** ^1^ Cereal Institute, Henan Academy of Agricultural Sciences, Zhengzhou, China; ^2^ State Key Laboratory of North China Crop Improvement and Regulation/Key Laboratory of Crop Growth Regulation of Hebei Province, College of Agronomy, Hebei Agricultural University, Baoding, China; ^3^ Key Laboratory of North China Water-saving Agriculture, Ministry of Agriculture and Rural Affairs, Baoding, Hebei, China

**Keywords:** blending controlled-release urea with conventional urea, deficit irrigation, summer maize, 13C-photosynthate distribution, nitrogen agronomic use efficiency, water use efficiency

## Abstract

**Introduction:**

High rainfall and excessive urea application are counterproductive to summer maize growth requirements and lower grain yield and water/nitrogen (N) use efficiency. The objective of this study was to determine whether ETc irrigation based on summer maize demand and reduced nitrogen rate in the Huang Huai Hai Plain increased water and nitrogen use efficiency without sacrificing yield.

**Methods:**

To achieve this, we conducted an experiment with four irrigation levels [ambient rainfall (I0) and 50% (I1), 75% (I2), and 100% (I3) of actual crop evapotranspiration (ET_c_)] and four nitrogen rates [no nitrogen fertilizer (N0), recommended nitrogen rate of urea (NU), recommended nitrogen rate of blending controlled-release urea with conventional urea fertilizer (BCRF) (NC), and reduced nitrogen rate of BCRF (NR)] in 2016–2018.

**Results:**

The results show that reduced irrigation and nitrogen rate reduced Fv/Fm, ^13^C-photosynthate, and nitrogen accumulation both in the kernel and plant. I3NC and I3NU accumulated higher ^13^C-photosynthate, nitrogen, and dry matter. However, ^13^C-photosynthate and nitrogen distribution to the kernel was decreased from I2 to I3 and was higher in BCRF than in urea. I2NC and I2NR promoted their distribution to the kernel, resulting in a higher harvest index. Compared with I3NU, I2NR increased root length density by 32.8% on average, maintaining considerable leaf Fv/Fm and obtaining similar kernel number and kernel weight. The higher root length density of I2NR of 40–60 cm promoted ^13^C-photosynthate and nitrogen distribution to the kernel and increased the harvest index. As a result, the water use efficiency (WUE) and nitrogen agronomic use efficiency (NAUE) in I2NR increased by 20.5%–31.9% and 11.0%–38.0% than that in I3NU, respectively.

**Discussion:**

Therefore, 75%ET_c_ deficit irrigation and BCRF fertilizer with 80% nitrogen rate improved root length density, maintained leaf Fv/Fm in the milking stage, promoted 13C-photosynthate, and distributed nitrogen to the kernel, ultimately providing a higher WUE and NAUE without significantly reducing grain yield.

## Graphical Abstract

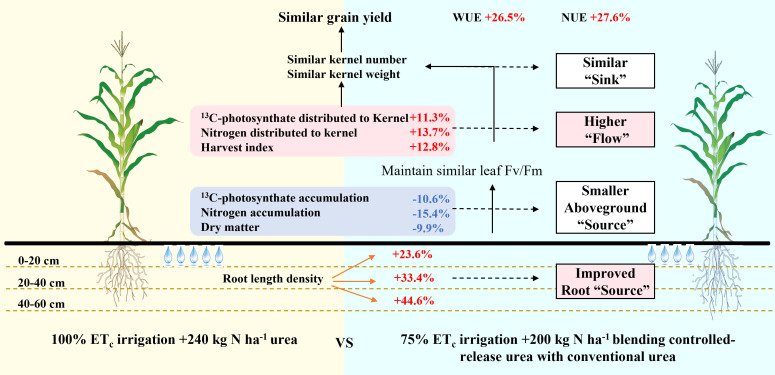

## 1 Introduction

Population expansion and climate change are generating water scarcity worldwide. As 70% of the fresh water supply is used by agriculture, water scarcity is a threat to the sustainability of agriculture (Mishra et al., 2021; Salehi, 2022). Increasing agricultural water use efficiency is a priority for food security and an effective way to mitigate water scarcity (Wang et al., 2019a). This problem is particularly serious in China, which hosts 6% of the world freshwater resources and feeds a significant amount of the world population.

**Figure 1:**
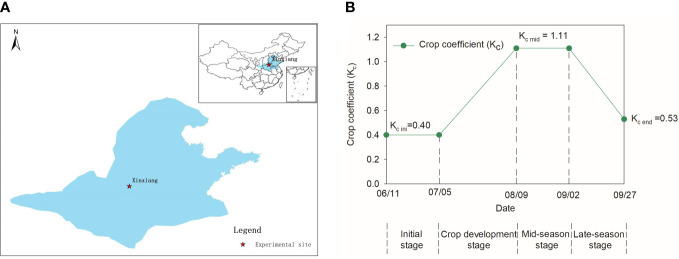
The experimental site in Huang-Huai-Hai maize region (blue area) of China **(A)** and the single crop coefficients K_c_ for the various development stages for summer maize in this experiment **(B)**.

With the greatest total output and acreage in China, maize is the most widely planted crop in the Huang-Huai-Hai Plain (**Figure 1A**), accounting for 35% of the national maize planting acreage and more than 40% of the corn grain output in China (Shu et al., 2021). Grown in the rainy season, summer maize received 310.0–536.4 mm of rainfall from 1961 to 2015 but only 115.5–166.0 mm of effective rainfall, which is significantly less than it requires (312.7–389.1 mm). Due to this misalignment between the rainfall and the maize’s water demand (Tuan et al., 2011), local farmers must irrigate their crops two or three times each year to increase the maize yield. Flood irrigation averaging 90–100 mm of water increases evaporation loss and nitrogen leaching, reducing water and nitrogen use efficiencies while polluting the environment (Guo et al., 2010; Eekhout et al., 2018; Yu et al., 2019; Hou et al., 2021).

Deficit irrigation maximizes water productivity and achieves water delivery equal to or better than full irrigation cultivation (Zhang, 2003b; Geerts and Raes, 2009; Comas et al., 2019; Sandhu et al., 2019). Deficit irrigation reduces soil evaporation and regulates leaf stomatal opening to reduce transpiration water loss, maintaining high photosynthetic efficiency (Ullah et al., 2019; Jovanovic et al., 2020). Understanding maize water requirements is the basis of deficit irrigation strategy. Preliminary research demonstrated that maize water requirements varied by variety, weather, and soil conditions and that all these variables should be addressed when making irrigation decisions (Peng et al., 2019; Masupha and Moeletsi, 2020; Mirhashemi and Panahi, 2021). Nevertheless, the current irrigation strategy is mainly based on the field capacity (Guo et al., 2015; Liu et al., 2022a), ignoring crop requirements and meteorological conditions. Local stress irrigation based on surface irrigation (i.e., border irrigation) has a high single irrigation volume (approximately 60–120 mm) but low irrigation frequency, which is performed by two irrigations at the sowing and flowering stages (Wang et al., 2020). The high irrigation level induces higher soil evaporation between plants and the water leachate (Srivastava et al., 2020; Yi et al., 2022). Currently, the most widely used conventional irrigation system is not compatible with advanced and efficient irrigation equipment, such as drip irrigation, sprinkler irrigation, or low-pressure pipe irrigation methods necessary to cover an area of 23,191×10^3^ ha, 30.1% of the total irrigated area in 2020 in China (Ministry of Water Resources, 2020). It is critical, therefore, to investigate modern agricultural irrigation systems that are geared to water conservation, high land efficiency, and labor efficiency.

The crop evapotranspiration (ET_c_) metric based on the FAO56 whitepaper is commonly used to make irrigation decisions, since it takes both crop requirements (growth phases) and climatic circumstances into account (Pereira et al., 2020). It also provides a precise and flexible irrigation schedule for an automatic or digital irrigation system, which is an excellent approach to cut labor expenses and water loss, and is accepted and employed by a growing number of farmers (Cancela et al., 2015). The majority of irrigation decision-making research has been conducted using models (Mancosu et al., 2016; López-Urrea et al., 2020), and the field performance of irrigation based on ET_c_ needs additional investigation. ET_c_ irrigation applied at 100% increases maize growth, net photosynthetic rate, and accumulation of dry matter (Guo et al., 2022). However, the effect of ET_c_ irrigation on photosynthetic transport, root length density, and water-saving potential of deficit ET_c_ irrigation on maize are currently unknown.

Numerous studies have demonstrated that soil moisture content and nitrogen availability have a complicated effect on crop yield (Sandhu et al., 2019). Irrigation that is appropriate for the soil could improve nitrogen use efficiency (NUE) by increasing nitrogen accumulation, translocation, and distribution (Yan et al., 2019). Blending controlled-release urea with conventional urea (BCRF) is both environment and economic friendly and has a wide range of applications (Vejan et al., 2021; Guo et al., 2022). Compared with urea, BCRF reduces N_2_O emissions (Lyu et al., 2021) and nitrogen leachate (Adu-Gyamfi et al., 2019), meets crop nitrogen demands (Zheng et al., 2020), and increases photosynthetic efficiency (Guo et al., 2022), crop yield, and NUE (Zheng et al., 2016; Zhu and Zhang, 2016; Vejan et al., 2021). As a result, the optimal nitrogen rate for BCRF and ET_c_ irrigation levels may differ from the optimal nitrogen rate under standard irrigation and nitrogen management methods. While earlier research was focused on crop grain yield, water use efficiency (WUE), and NUE, the interplay between ET_c_ irrigation and BCRF on maize performance is less studied.

The purpose of this study was to (1) examine how reduced water and nitrogen input increase water and nitrogen use efficiency without compromising yield, (2) investigate the effect of deficit irrigation based on ET_c_ and BCRF on photosynthate accumulation and distribution, and (3) investigate the interaction between deficit ET_c_ irrigation and BCRF-dependent reduced nitrogen rates on WUE and NUE, to lay the groundwork for more precise irrigation and fertilization maize crop management strategies.

## 2 Materials and methods

### 2.1 Site and weather description

**Figure 2:**
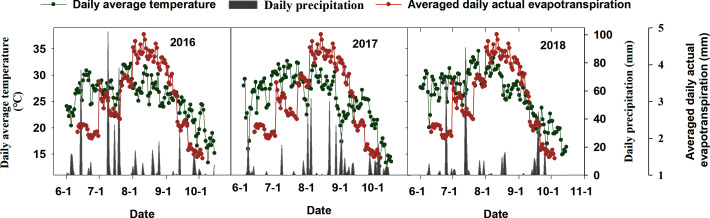
Daily mean temperature (°C) and precipitation (mm) during the summer maize season in 2016–2018 and the average daily actual evapotranspiration from 1983 to 2013.

From 2016 to 2018, studies were conducted in the Henan Academy of Agricultural Sciences experimental base in Yuanyang, Henan Province, China (113°42′28.7′′N, 35°0′13.3′′E), 78 m above sea level). The regional climate is subhumid, warm temperate, continental, monsoon, and features four distinct seasons. This is a typical location on the Huang-Huai-Hai Plain (**Figure 1A**). The ET_c_ was calculated using weather data from 1983 to 2013 received from China’s National Meteorological Information Center (climate data, **Figure 2**). Precipitation totaled 349.4, 193.6, and 239.4 mm during the maize growth period in 2016, 2017, and 2018, respectively.

The regional soil is fluvo-aquic, with 13.44 g kg^−1^ organic matter, 73.8 g kg^−1^ available N, 50.2 mg kg^−1^ available phosphate, and 134.5 mg kg^−1^ available potassium under a rainproof shelter and 13.86 g kg^−1^ organic matter, 86.13 g kg^−1^ available N, 53.5 mg kg^−1^ available phosphate, and 146.0 mg kg^−1^ available potassium in the field.

### 2.2 Experimental design

The experiment used a randomized complete block design with 16 treatments (4 irrigation levels and 4 nitrogen fertilizer treatments) in triplicate. The four irrigation levels were ambient rainfall (I0), 50% ET_c_ (I1), 75% ET_c_ (I2), and 100% ET_c_ (I3). The four nitrogen fertilizer treatments were as follows: no fertilizer (N0), urea with the recommended nitrogen rate (240 kg N ha^−1^, NU), BCRF with the recommended nitrogen rate (240 kg N ha^−1^, NC), and BCRF with a reduced nitrogen rate (200 kg N ha^−1^, NR).

To avoid the potential impact of unforeseen rainfall on the experiment, maize, the experiment of I1, I2, and I3 was performed in microplots beneath an autonomously triple-folding rainproof shelter in 2016 and 2017. When it rained, the rainproof shelter was opened to cover the plots and keep the rain off. At other times, the rainproof shelter was stored adjacent to the experimental plots in an unoccupied location. The plants in I0 treatment received only rainfall with no supplemental irrigation from 2016 to 2018. In 2018, the experiment was conducted in a field 300 m away. That is, the water input in I0 was only rainfall in three maize seasons; the water input in I1, I2, and I3 in 2016 and 2017 under rainproof shelter was only irrigation; while water input in I1, I2, and I3 in field in 2018 was the sum of irrigation and rainfall.

The microplot was 2.9 m × 1.9 m in size, and the field plot was 4.2 m × 6.7 m in size. In 2016, 2017, and 2018, summer maize (Zhengdan 309, a national maize variety suitable for harvesting grain mechanically) was planted on June 16, June 16, and June 17 and harvested on September 30, October 3, and October 5, respectively. A precision irrigation equipment irrigated the maize. The previous crop, winter wheat, was irrigated fully and fertilized with no nitrogen to achieve identical soil moisture and nitrogen concentrations between microplots before planting maize. The maize was planting in 75,000 plans ha^−1^ with 60 cm plant row spacing. Pest, disease, and weed control strategies were similar to those used regionally.

#### 2.2.1 Irrigation regime

Actual crop evapotranspiration (ET_c_) was determined by the following formula:


$${\text{ET}}_{\text{c}}={\text{ET}}_{0}\times {\text{K}}_{\text{c}}$$

ET_0_ is averaged daily reference evapotranspiration (mm), calculated using daily meteorological data from 1983 to 2013 (from the National Meteorological Information Center of China) with the ET_0_ calculator (Food and Agriculture Organization (FAO56). K_c_ is the crop coefficient, determined by the FAO56 guidelines. The lengths of the crop development phases for the initial stage, development stage, mid-season stage, and late-season stage were 26, 34, 24, and 20 days, respectively, according to FAO56. The K_c_ values for the initial stage, mid-season stage, and end of the late-season stage were 0.4, 1.11, and 0.53, respectively (**Figure 1B**).

The irrigation amounts for I1, I2, and I3 were calculated as follows:


$$\text{I}1=50\%{\text{ ET}}_{c}-{\text{P}}_{e}$$

$$\text{I}2=75\%{\text{ ET}}_{\text{c}}-{\text{P}}_{\text{e}}$$

$$\text{I}3=100\%{\text{ ET}}_{\text{c}}-{\text{P}}_{e}$$

P_e_ is the effective precipitation amount (mm), and ET_c_ is the actual crop evapotranspiration. P = 0 for I1, I2, and I3 in 2016 and 2017 under rain shelter when it rains. P_e_ was calculated as P_e_=a×P, in which a was 0, 1.0, and 0.75 when the precipitation<5 mm, 5 mm≤precipitation ≤ 50 mm, and precipitation >50 mm, respectively.

For both the microplot and field tests, 44.1 mm of water was irrigated after sowing to enable maize seedling emergence. Aside from the sowing irrigation, the plants were irrigated at the V6, V12, VT, R2, and R4 stages. The irrigation amounts and the precipitation levels at different irrigation levels in 2016–2017 and 2018 are shown in **Tables 1**, **2**.

**Table 1:** Irrigation volume of different irrigation levels at various summer maize growth stages in 2016 and 2017.

Item	Growth stage	Irrigation amount (mm)			
		I0	I1	I2	I3
Irrigation amount (mm)	Sowing	44.1	44.1	44.1	44.1
	V6	0	20.9	31.4	41.8
	V12	0	27.4	41.1	54.8
	VT	0	30.8	46.2	61.6
	R2	0	35.6	53.4	71.2
	R4	0	31.1	46.6	62.1
Irrigation total		44.1	189.9	262.8	335.6
Precipitation *	2016	349.4	0	0	0
	2017	193.6	0	0	0
Irrigation +precipitation	2016	393.5	189.9	262.8	335.6
	2017	237.7	189.9	262.8	335.6

^*^Precipitation in the whole growing season.

**Table 2:** Precipitation and irrigation volume of different irrigation levels at various summer maize growth stages in 2018.

Growth stage	Irrigation amount (mm)				Precipitation * (mm)
	I0	I1	I2	I3	
Sowing	44.1	44.1	44.1	44.1	87.6
V6	0	0	0	0	143.4
V12	0	0	0	0	15.6
VT	0	15.2	30.6	46	31.8
R2	0	3.8	21.6	39.4	111.4
R4	0	0	0	0	0
Total irrigation	44.1	63.1	96.3	129.5	–
Irrigation +precipitation	433.9	452.9	486.1	519.3	389.8

^*^Precipitation during the previous growth stage.

#### 2.2.2 Fertilizer management

Four fertilizer treatments were tested: (i) a quick-release urea (46% N) with a 240 kg N kg^−1^ application rate, (ii) a BCRF (26% N, 10% P_2_O_5_, and 9% K_2_O; Kingenta; controlled-release fertilizer: quick-acting fertilizer = 1:1) with a 240 kg N kg^−1^ application rate (NC), (iii) a BCRF with a 200 kg N kg^−1^ application rate (NR), and (Srivastava et al.) no nitrogen fertilizer (N0). All plots had the same phosphate and potassium rates of 150 kg P_2_O_5_ kg^−1^ and 120 kg K_2_O kg^−1^, respectively. Calcium superphosphate (12.0% P_2_O_5_) and potassium chloride (52.0% K_2_O) fertilizers were used to compensate for the deficiency of phosphate and potassium. As a basal fertilizer, 100% BCRF, 50% urea, 100% calcium superphosphate, and potassium chloride fertilizer were applied. At the tasseling stage (VT), urea (50%) was applied as a topdressing fertilizer. Winter wheat was planted prior to maize, and it was fertilized with nitrogen-free fertilizer to maintain a same soil nitrogen content prior to planting maize.

### 2.3 Sampling, measurements, and calculations

#### 2.3.1 Meteorological data

Meteorological data, including rainfall, temperature, air humidity, and wind speed, were obtained automatically at a station 200 m from the trial site.

#### 2.3.2 Labeling of selected plants with ^13^CO_2_

Six representative plants from each plot were selected and labeled with ^13^CO_2_ on silking stage. Ear leaves of each plant were covered in 0.1-mm thick mylar plastic bags, which permitted up to 95% of natural sunlight intensity. After sealing the bags at the base with Plasticine, 50 ml of ^13^CO_2_ was injected. After 60 min, the ^13^CO_2_ in each bag was extracted using a KOH washer to remove any residual radioactive ^13^CO_2_, and the plastic bag was removed (Liu et al., 2015).

#### 2.3.3 Dry matter, ^13^C-photosynthate distribution ratio, and nitrogen distribution ratio among plant organs

The labeled plants were collected at physiological maturity and dissected into leaves, stem, sheath, ear bract, cob, and grain. The harvest index was calculated as the ratio of grain dry matter to total plant dry matter. All separated components were oven-dried to a constant weight at 80°C, weighed to determine dry matter (g plant^−1^), and then milled into a powder. Subsamples of 4 mg were used to determine the isotopic abundance using an Isoprime 100 instrument (Isoprime 100, Cheadle, UK). Subsamples were digested using an H_2_SO_4_–H_2_O_2_ method (Thomas et al., 1967), and total nitrogen was measured using a continuous flow autoanalyzer (AA3, SEAL Analytical, Germany). The ^13^C-photosynthate accumulation (^13^C-AC) and distribution ratio (^13^C-DR) among different plant organs at physiological maturity (%/plant) and the nitrogen distribution accumulation (N-AC) and distribution ratio (N-DR) were calculated.

#### 2.3.4 Ear leaf Fv/Fm and root length density

Using a continuous excitation fluorometer Pocket Plant Efficiency Analyzer (PEA, Hansatech, UK), the Fv/Fm of six representative ear leaves at the silking stage (R1) and milk stage (R3) were determined under dark conditions for 15 min. Three maize plants in each treatment were selected to evaluate root length density at the anthesis stage. In the 0–60-cm soil layer, a block of soil surrounding the plant (60 cm long, 22 cm wide, 20 cm deep; 26,400 cm^3^) was removed for each plant sample. The root samples were carefully cleaned of non-root material. Root lengths were measured using WinRHIZO (Regent Instruments Inc., Canada) after the fresh roots were scanned using an Epson Perfection V800 scanner. Root length density was calculated by dividing root length by soil volume (26,400 cm^3^).

#### 2.3.5 Yield and harvest index

All ears in each plot were collected at the physiological maturity to investigate the yield and yield components. For each harvested ear, the kernel number per plant (KNP) was counted. Three 1,000-kernel samples were oven-dried at 80°C to a consistent weight and weighed to determine the kernel weight (KW). To determine grain yield, all kernels were air-dried, and grain yield was expressed at 14% moisture content.

#### 2.3.6 Nitrogen agronomic use efficiency and water use efficiency

The nitrogen agronomic use efficiency (NAUE, kg kg N^−1^) was calculated as follows:


$$\text{NAUE}=\left({\text{Y}}_{\text{fertilizer N}}-{\text{Y}}_{\text{N}0}\right)/\text{nitrogen rate}$$

Y _Fertilizer N_ is the grain yield (kg ha^−1^) for the nitrogen fertilizer treatment (NC, NR, and NU), and Y_N0_ is the grain yield for the N0 treatment. The nitrogen rate was the nitrogen fertilizer applied for the nitrogen fertilizer treatment.

The water use efficiency (WUE, kg m^-3^) was calculated as follows:


WUE=Y/ET

ET=ρW+I+P−R−D

Y is the grain yield (kg ha^−1^), ET is evapotranspiration (mm), ρW is variation in soil water storage in the 0–100-cm soil layer between planting and maturity, I is the water input (the sum of irrigation, mm), and P is precipitation (i.e., rain in our study). R is the water lost to runoff from the ground surface, which was zero in this experiment due to the borders for each plot in both canopied (2016–2017) and in open-field (2018) microplots. D is deep percolation from the soil, which was ignored due to the low amount of irrigation in 2016 and 2017.

### 2.4 Statistical analyses

Data were analyzed using Microsoft Excel 2016 and mapped using Sigma Plot 10.0 and Origin 2021. The SAS software system for Windows 9.0 (SAS Institute, Cary, NC, USA) was used to perform analyses of variance (ANOVAs). An ANOVA was performed among all the irrigation and nitrogen treatments for grain yield, yield components, dry matter, harvest index, WUE, and NAUE (*p*<0.05). The multiple comparison procedure (SSR) test with Bonferroni correction for all treatments was used for multiple comparisons.

## 3 Results

### 3.1 Yield, dry matter, and harvest index

Irrigation, nitrogen fertilizer, and their interaction all had a substantial impact on the kernel number per ear, kernel weight, grain yield, dry matter, and harvest index (**Table 3**). The yield and dry matter increased with irrigation level, but there was no significant difference between the yields of I2 and I3. Compared with I3, I0 and I1 significantly decreased the kernels number per ear and kernel weight. I2 had a lower KNP but higher kernel weight than I3. For the same irrigation levels in I2 or I3, the kernels number per ear and kernel weight of three nitrogen fertilizers show the trend that NC>NR>NU, but there was no significant difference either between NC and NR or between NR and NU.

**Table 3:** Effect of irrigation and nitrogen fertilizer on the grain yield, yield components, dry matter, and harvest index for summer maize in 2016–2018.

Treatment	2016					2017					2018				
	KNP	KW(g)	Yield(kg ha^-1^)	Dry matter(kg ha^-1^)	HI(%)	KNP	KW(g)	Yield (kg ha^-1^)	Dry matter (kg ha^-1^)	HI(%)	KNP	KW(g)	Yield (kg ha^-1^)	Dry matter (kg ha^-1^)	HI(%)
I0N0	264fg	263.3f	4430.6i	9657.4g	45.9e	267.3e	246.3d	4196.4h	9207.5h	43.3e	297.3g	258.8e	4899.9f	11765.9j	41.6g
I0NC	344bcd	303bc	6645.4d	13295.5bc	50.1bcd	314c	287.5b	5753.9d	10612.3def	51.5ab	384.7e	289.8c	7106d	16015.8f	44.4ef
I0NR	332cde	298.5c	6317.7e	12734.7cd	49.7bcde	309cd	287.4b	5660.5de	10440efg	51.5ab	391.7e	280.8cd	7012.2d	15328.4gh	45.8de
I0NU	324de	301.9bc	6231.9e	13099.9c	47.6de	310cd	288.6b	5703.7de	10902.7de	49.7bc	390.7e	282.9cd	7045.3d	16072.7f	43.8f
I1N0	240g	239.8g	3668.8j	7464h	49.3bcde	267.7e	246.4d	4202.4h	9074.5h	44.0e	363.3f	275.3d	6378.1e	13935.2i	45.7de
I1NC	332cde	279.1d	5905.2f	11326.4e	52.1bc	314.7c	283.2b	5680de	10619.3def	50.8ab	446.0c	313.6b	8914.4c	19478.7e	45.8de
I1NR	316e	273.9de	5517.3g	10699.1f	51.6bc	310.3cd	280.5b	5548.6ef	10267.9fg	51.3ab	444.7c	315.3b	8934.4c	19027e	47cd
I1NU	308e	272.2de	5344.2g	10966.8ef	48.8cde	306.3cd	281.1b	5486f	10957d	47.6cd	448.7c	305.6b	8738.3c	19419.3e	45ef
I2N0	276f	272de	4781.7h	9359.1g	51.1bcd	296.7d	265.1c	5012.6g	9965g	47.8cd	395.8e	282.1cd	7116.7d	14920.6h	47.7bc
I2NC	372ab	314.4a	7454.7a	13198.4c	56.5a	348.3ab	308.1a	6841.1ab	12604.7b	51.5ab	482.9b	338.7a	10427.5ab	21456.8bc	48.6bc
I2NR	367.3ab	308.9ab	7233b	12369d	58.5a	344.3b	307.3a	6743.7b	12090.9c	53.0a	480b	334.8a	10244.8ab	20261d	50.6a
I2NU	346bcd	311.2a	6929.7c	13003.8c	52.7b	336.3b	307a	6581.5c	12445.2bc	50.2b	482.3b	330.1a	10149.4b	21144.8c	48bc
I3N0	280f	268.8ef	4796.7h	9819.7g	48.9bcde	302cd	259c	4984.4g	10000.2g	47.4cd	411.6d	274.8d	7213.3d	15728.2fg	45.9de
I3NC	376.7a	311.6a	7479.6a	14378.2a	52bc	360a	303.6a	6966.2a	13472.3a	49.1bcd	500.2a	332.6a	10605.1a	22208.9a	47.7bc
I3NR	368ab	307.3ab	7193.1b	13841.7ab	52bc	350ab	301a	6715.8bc	12471.3bc	51.2ab	490.1ab	330.4a	10324ab	21099.1c	48.9ab
I3NU	354.7abc	310.8a	7001.1c	14338.9a	49.1bcde	340b	303.6a	6580.1c	13362.5a	46.8d	490.7ab	330.1a	10326.5ab	21667.6b	47.7bc
Two-factor ANOVA															
F value (Irrigation)	24.9**	171.2**	513.6**	165.3**	21.7**	70.7**	77.5**	505.2**	193.3**	7.5**	349.9**	111.1**	465.4**	1018**	50.8**
F value (Nitrogen)	91.0**	241.4**	1243.3**	367.8**	13.6**	91.4**	245.5**	920.2**	206.8**	54.4**	280.3**	134.5**	426.3**	1302.4**	16.0**
F value (Irrigation ×Nitrogen)	0.3ns	0.5ns	5.4**	3.6**	4.9**	0.4ns	3.4**	4.4**	7.8**	3.7**	1.1ns	4.1**	4.0**	10.6**	3.5**

KNP, KW and HI were kernel number per ear, 1000-kernel weight, and harvest index, respectively. Irrigation × Nitrogen was the interaction of irrigation and nitrogen fertilization. The value underlined was significantly higher than other treatments. Different letters in the same column indicate significant differences at the 0.05 level. *: significant at P ≤ 0.05; **: significant at P ≤ 0.01, NS: not significant at P ≤ 0.05.

The harvest index rose with irrigation level, then subsequently fell with I2 representing the peak. The HI was highest in NR and lowest in NU. Over three seasons, the plants in I2NR produced a similar yield to those in I3NC treatments despite having a lower dry matter but a higher harvest index.

### 3.2 The ^13^C-photosynthate/nitrogen accumulation and distribution ratio

**Figure 3:**
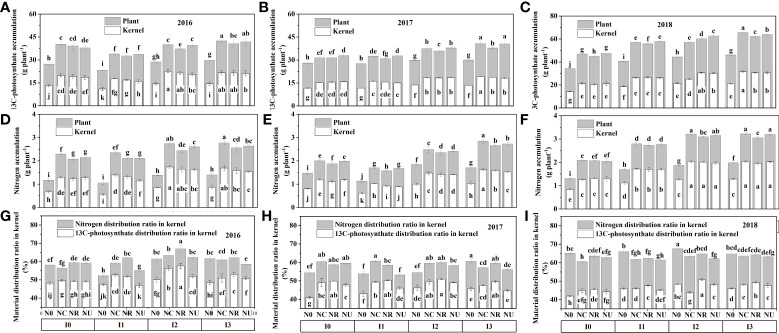
The effect of irrigation and nitrogen fertilizer on the 13C-photosynthate accumulation **(A–C)**, nitrogen accumulation **(D–F)**, and 13C-photosynthate distribution ratio and nitrogen distribution ratio **(G–I)** in the kernel at physiological maturity. The relative data in 2016 is shown in panels **(A, D, G)**; in 2017, panels **(B, E, H)**; and in 2018, **(C, F, I)**. Different letters in the same figure indicate significant differences at the 0.05 level.

The irrigation, nitrogen fertilizer, and their interaction had a significant effect on ^13^C-photosynthate/nitrogen accumulation and distribution ratio (**Supplementary Table S1**). The ^13^C-AC and N-AC of the plant and kernel increased with the irrigation level from I1 to I3, and nitrogen rate from 0 to 240 kg N ha^−1^ in the three growth seasons (**Figure 3**). Although there was no discernible difference between NU and NC in 2016, I3NC had the greatest ^13^C-AC and N-AC of the plant and kernel, followed by I3NU, I2NC, and I2NU.

^13^C-DR and N-DR both increased when irrigation level increased from I1 to I2, while they decreased from I2 to I3. The ^13^C-DR of N0 was higher than that of NC, NU, and NR. Whereas the N-DR of N0 was lower than NC, NU, and NR. For the nitrogen fertilized treatments, the ^13^C-DR and N-DR in the kernels was higher in I2NR in 2016 and 2017 with no significant difference between I2NC, I2NR, and I3NC; that is, the BCRF fertilizer promoted the distribution of ^13^C-photosynthate and nitrogen to the kernel. Thus, a higher ^13^C-AC and N-AC were obtained in the I3NC and I3NU treatments, and the highest ^13^C-DR and N-DR in the kernel were obtained in I2NR.

### 3.3 Root length density

**Figure 4:**
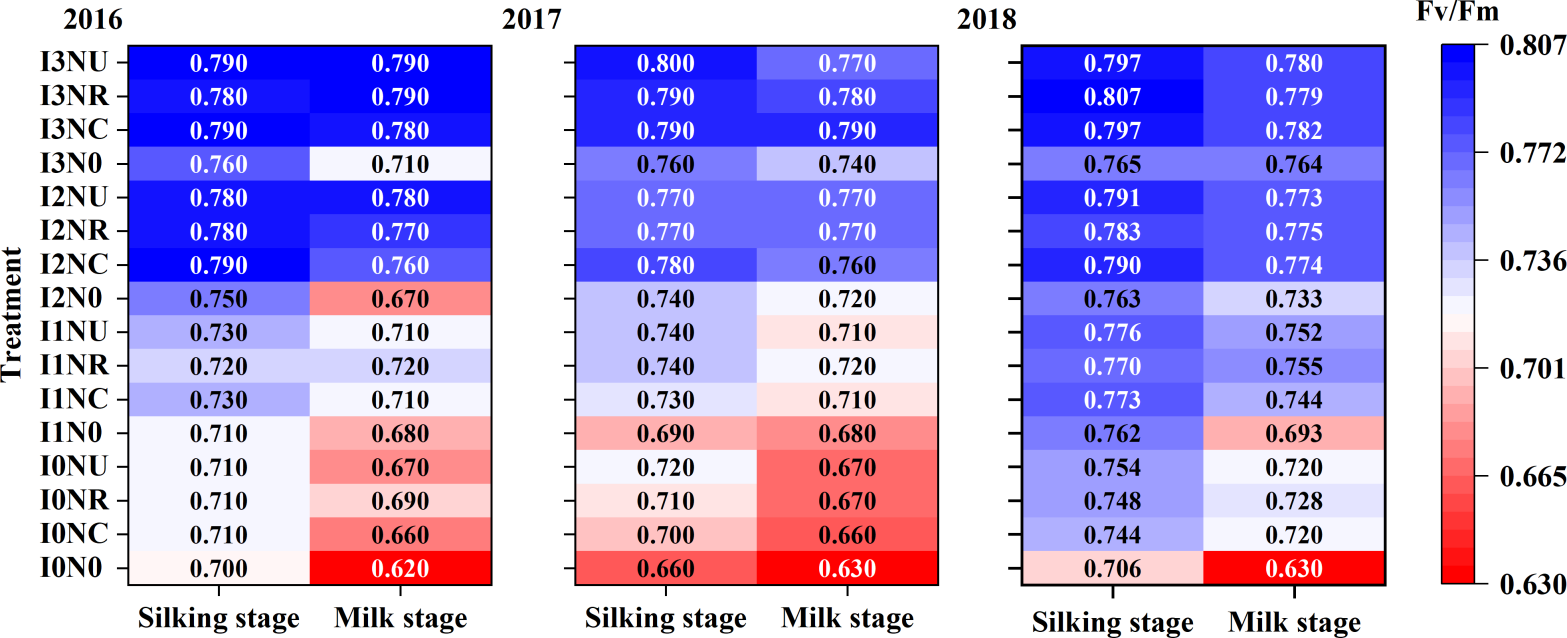
Effect of irrigation and nitrogen fertilizer on Fv/Fm at the silking stage and milk stage in 2016, 2017 and 2018.

There was a significant effect of irrigation treatment and nitrogen fertilizer on root length density (**Supplementary Table S1**). The root length density of 0–20 cm, 20–40 cm, and 40–60 cm accounted for 68.4%–76.4%, 18.8%–24.3%, and 4.3%–7.5% of total root length density (**Figure 4**). Root length density of either soil layer was increased with irrigation level from I0 to I2 and decreased from I2 to I3. In 0–60 cm, root length density was higher in NC and NR than in NU under the same irrigation level, while the difference was not statistically significant. There was no significant difference between root length density of NR and NC, except that NR was higher than NC under I2 and I1 irrigation levels at 40–60-cm soil layer. The I2NR treatment achieved the maximum root length density in the 0–20-cm and 40–60-cm soil layers, whereas I2NR, I2NC, and I2NU treatments achieved the highest root length density in the 20–40-cm soil layer. These results imply that (1) compared with urea, BCRF could improve maize root length density, and (2) Root length density of I2NR in the 0-60-cm soil layer, particularly in the 40-60-cm layer, was much higher than that of I3NC or I3NR.

### 3.4 Maximum photochemical efficiency of photosystem II (Fv/Fm)

**Figure 5:**
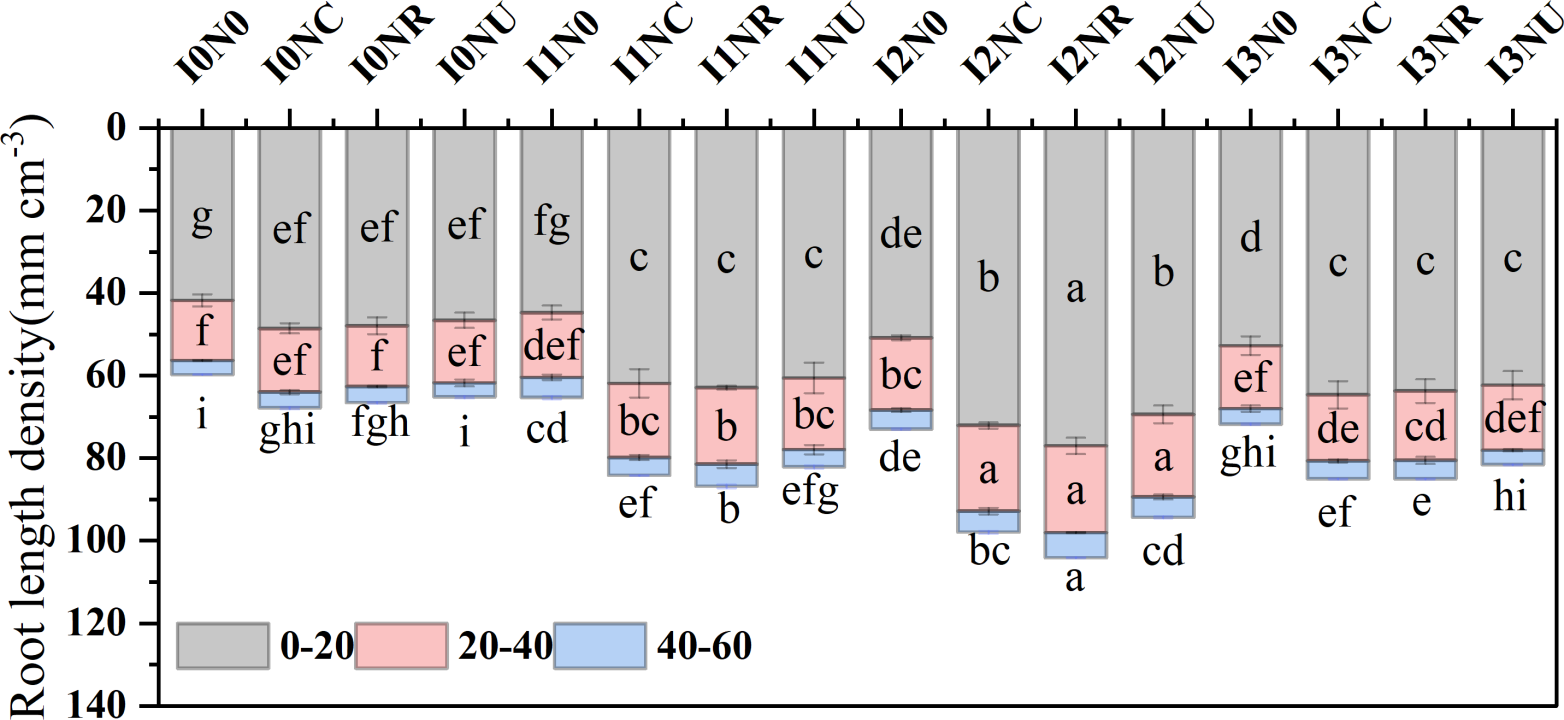
Effect of irrigation and nitrogen fertilizer on root length density. Different letters on the gray, pink, and blue histograms indicate significant differences at the 0.05 level.

Irrigation and nitrogen fertilizer management both had a significant impact on the Fv/Fm ratio (**Supplementary Table S1**). There was no significant difference between the Fv/Fm of NC, NR, and NU treatments, but the Fv/Fm for NC, NR, and NU were significantly higher than that for N0 (**Figure 5**). With the application of nitrogen fertilizer, the I3 treatment had the highest Fv/Fm at the silking stage growth stage, followed by I2, I1, and I0. The Fv/Fm decreased significantly from silking stage to the milk stage, with average decreases of 6.2%, 3.1%, 2.6%, and 1.7% for I3, I2, I1, and I0, respectively. At the milk stage, Fv/Fm values were increased with irrigation level, but there was no significant difference between Fv/Fm values of I3 and I2. It may be concluded that irrigation at I3 and I2 benefited for maintaining a higher Fv/Fm of the ear leaves.

### 3.5 Evapotranspiration and water use efficiency

**Figure 6:**
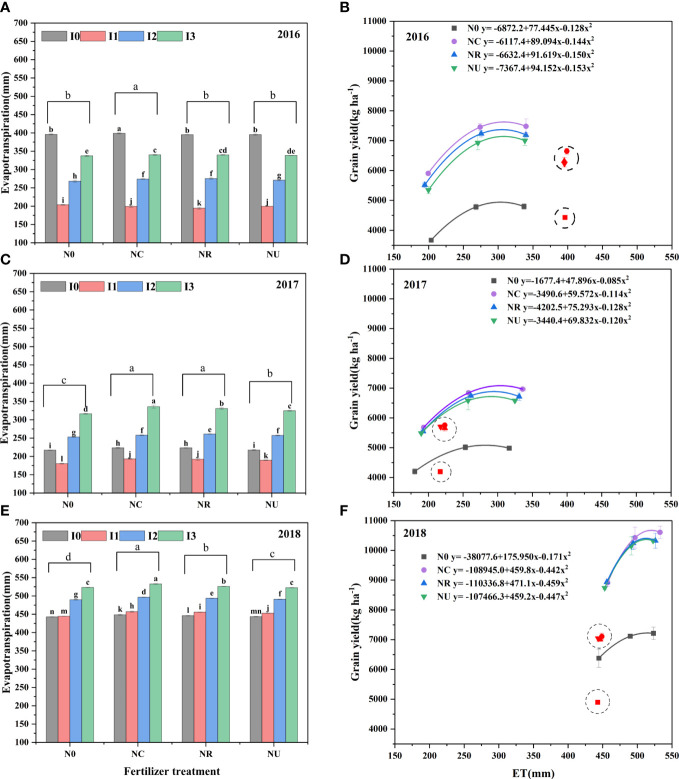
Effect of irrigation and nitrogen fertilizer on evapotranspiration and the relationship between grain yield and evapotranspiration. The data of I1, I2, and I3 were used to fit curves. Different letters on the gray, pink, and blue histograms indicate significant differences at the 0.05 level **(A, C, E)**. The curve with lines in blank, purple, blue, and green color was fitted with data of N0, NC, NR, and NU, **(B, D, F)** respectively.

Along with the amount of rainfall, ET was highest in 2018 (442.8–532.8 mm) and lowest in 2017 (180.4–336.0 mm) (**Figure 6**). Both irrigation and nitrogen fertilizer and their interaction had an impact on ET, but the effect of irrigation was higher than that of nitrogen fertilizer. The ET was increased with irrigation or rainfall showing increased from I1 to I3 (**Figure 6**). The ET of nitrogen fertilizer treatments was highest in NC, followed by NR and NU, and was lowest in N0 (**Figure 6**). Maize grain yield showed a parabolic trend of opening downward as ET increased. The WUE was highest obtain in I1 in 2016 and 2017 but highest in I2 in 2018 (**Table 4**). Similar to yield, the WUE was highest in NC, but the difference between NC with NR or NU was significant in 2016.

**Table 4:** Effect of irrigation and fertilizer treatment on water use efficiency and nitrogen agronomic use efficiency for summer maize in 2016–2018.

Treatment	WUE(kg m^-3^)			NAUE (kg kg N^-1^)		
	2016	2017	2018	2016	2017	2018
I0N0	1.12j	1.94h	1.11f			
I0NC	1.67h	2.57bcd	1.58d	9.2b	6.5d	9.0f
I0NR	1.60h	2.54d	1.57d	9.5b	7.3bc	11.8cde
I0NU	1.58h	2.62bc	1.59d	7.5cd	6.3d	9.8ef
I1N0	1.80g	2.33e	1.44e			
I1NC	2.96a	2.94a	1.95c	9.3b	6.2d	11def
I1NR	2.84b	2.89a	1.96c	9.2b	6.7cd	13.3bc
I1NU	2.68c	2.89a	1.93c	7.0d	5.3e	10.2ef
I2N0	1.79g	1.98gh	1.45e			
I2NC	2.72c	2.65b	2.10a	11.2a	7.6b	13.8abc
I2NR	2.63cd	2.58bcd	2.07ab	12.2a	8.7a	15.6a
I2NU	2.56d	2.56cd	2.07ab	8.7bc	6.5d	12.6bcd
I3N0	1.43i	1.58i	1.38e			
I3NC	2.21e	2.08f	1.99bc	11.2a	8.3a	14.1ab
I3NR	2.12ef	2.03fg	1.96c	12.0a	8.6a	15.6a
I3NU	2.07f	2.03fg	1.98c	9.3b	6.6d	13bcd
Irrigation(I)						
I0	1.49d	2.42b	1.46c	8.7b	6.7b	10.2c
I1	2.57a	2.76a	1.82b	8.5b	6.1c	11.5b
I2	2.42b	2.44b	1.92a	10.7a	7.6a	14.0a
I3	1.96c	1.93c	1.83b	10.8a	7.8a	14.2a
Nitrogen treatment(N)						
N0	1.53d	1.96c	1.35b	10.2a	7.1b	12.0b
NC	2.39a	2.56a	1.90a	10.7a	7.8a	14.1a
NR	2.30b	2.51b	1.89a	8.1b	6.2c	11.4b
NU	2.22c	2.53ab	1.89a			
F values						
I	771.18**	713.97**	202.7**	20.8**	41.9**	25.8**
N	493.37**	495.9**	375.87**	35.4**	57.1**	17.3**
I×N	11.03**	3.23**	1.81ns	3.0*	3.8*	0.5ns

WUE and NAUE were water use efficiency (kg m^-3^) and nitrogen agronomic use efficiency (kg kg N^-1^), respectively. Different letters in the same column indicate significant differences at the 0.05 level. *: significant at P ≤ 0.05; **: significant at P ≤ 0.01, NS: not significant at P ≤ 0.05.

### 3.6 Nitrogen use efficiency

Irrigation and nitrogen fertilizer management have a considerable impact on NAUE (5.3–15.6 kg N^−1^) (**Table 4**). The NAUE increased with irrigation level from I1 to I2, but there was no significant difference between I2NC, I2NR, I3NC, and I3NR in 2017. The NAUE of the NC and NR was significantly higher than the NU by 15.25% and 27.20% averagely, where there was no significant difference between the NAUE of NC and NR.

### 3.7 Correlation analysis

**Figure 7:**
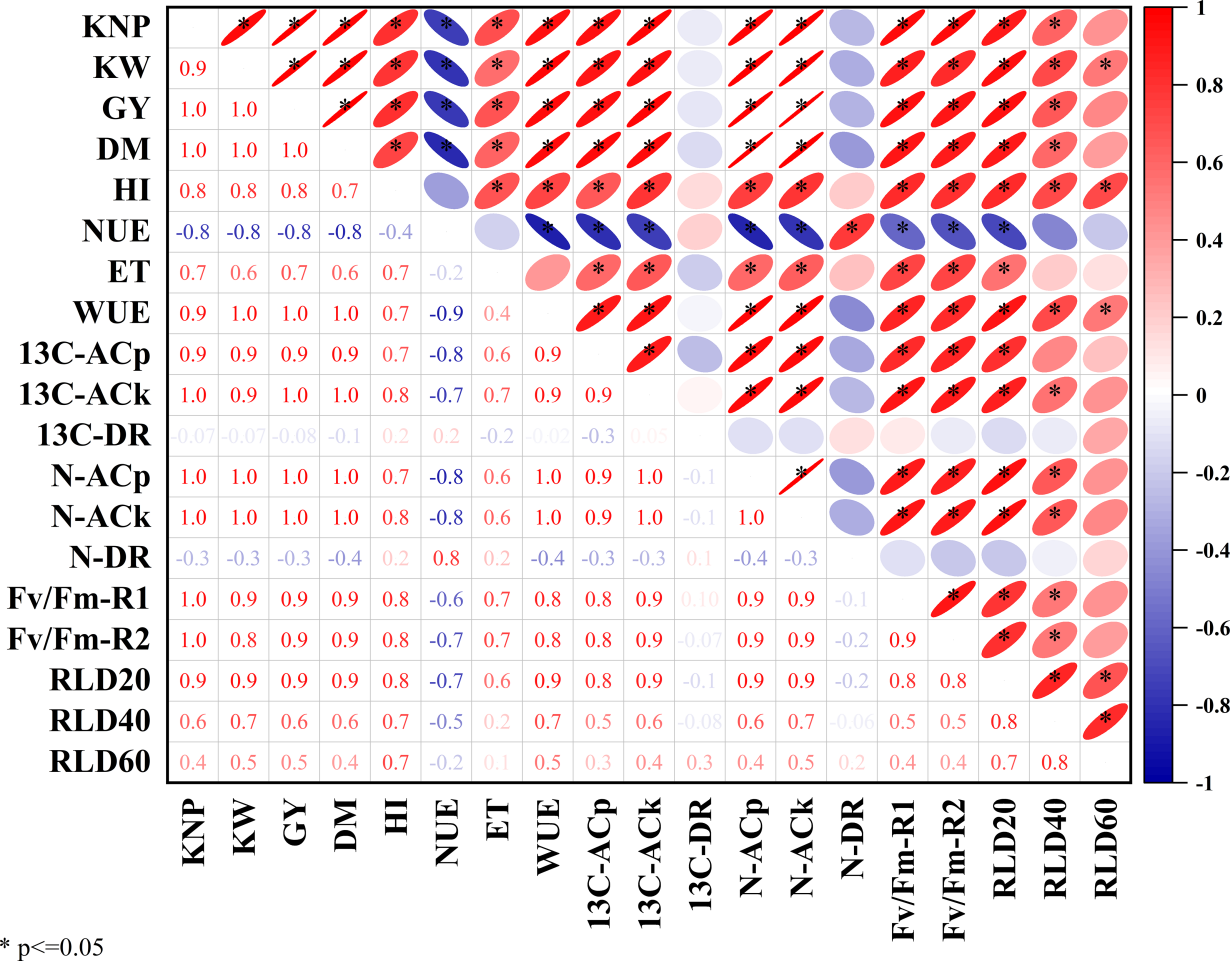
Correlation coefficients between maize grain yield, dry matter, ^13^C-photosynthate and nitrogen accumulation and distribution characteristics, leaf maximum photochemical efficiency of photosystem II, root length density, and nitrogen and water use efficiency. KNP, kernel number per ear; KW, kernel weight; GY, grain yield; NI, harvest index; NUE, nitrogen agronomic use efficiency; ET, evapotranspiration; WUE, water use efficiency; 13C-ACp, 13C-photosynthate accumulation in plant; 13C-ACk, 13C-photosynthate accumulation in kernel; 13C-DR, 13C-photosynthate distribution ratio in kernel; N-ACp, nitrogen accumulation in plant; N-ACk, nitrogen accumulation in kernel; N-DR, nitrogen distribution ratio in kernel; Fv/Fm-R1 and Fv/Fm-R2, maximum photochemical efficiency of photosystem II (Fv/Fm) in silking stage and milk stage, respectively; RLD20, RLD40 and RLD60, root length density for 0-20cm, 20-40cm and 40-60cm, respectively.

**Figure 8:**
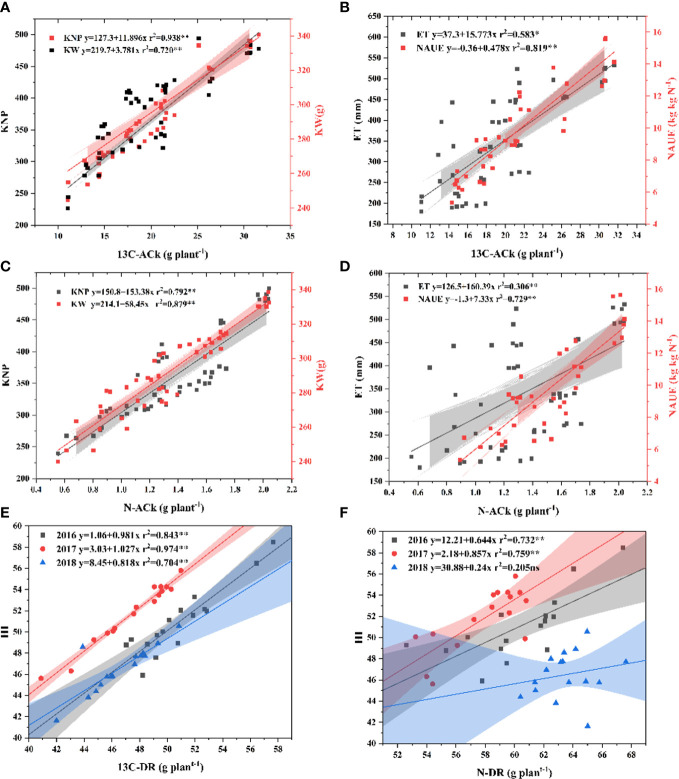
Scatterplots of kernel number per spike (KNP), kernel weight (KW), evapotranspiration (ET), and nitrogen agronomic use efficiency (NAUE) versus ^13^C-photosynthate accumulation in kernel (^13^C-ACk) **(A**, **B)** and nitrogen accumulation in kernel (N-ACk) **(C, D)**, ^13^C-photosynthate distribution ratio in kernel (^13^C-DR), and nitrogen distribution ratio in kernel (N-DR) versus harvest index (HI) **E, F)**, respectively. The colored areas indicate the 95% confidence intervals of the fitted curves.

Correlation analysis (**Figure 7**) revealed that the ^13^C-AC, N-AC, Fv/Fm, and root length density were all significantly and positively linked with grain yield, KNP, 1,000 KW, and WUE (*p*<0.05). The ^13^C-AC and N-AC of kernel were linearly related to kernel number per kernel, kernel weight, ET, and NAUE (**Figure 8**). The harvest index was increased linearly with the increase in ^13^C-DR and N-DR of kernel, except harvest index versus N-DR in 2018.

## 4 Discussion

### 4.1 Effect of irrigation and nitrogen fertilizer on ^13^C-photosynthate and nitrogen accumulation and distribution and harvest index

Maize grain yield is determined by dry matter and harvest index. The accumulation of dry matter is mostly determined by photosynthesis production, while the harvest index is primarily determined by the partitioning of photosynthate and biomass to kernels (Sinclair, 1998; Allison, 2010). ^13^C-labeled CO_2_ is an effective method for studying the accumulation and distribution characteristics of photosynthetic assimilate (Nouchi et al., 1995; Tremblay et al., 2012; Ren et al., 2021). ^13^C-photosynthate allocation to grain is positive for kernel weight and grain yield (Liu et al., 2015; Ren et al., 2021). In this study, both ^13^C-photosynthate and nitrogen accumulation in the kernel was positively related to kernel number per spike, kernel weight, and grain yield. Their distribution ratio to the kernel were found to be linearly related to harvest index. Increasing the harvest index was the primary strategy to increase maize grain yield at lower yield levels (<15 Mg ha^−1^) (Liu et al., 2020). Thus, management that increases the accumulation and distribution of ^13^C-photosynthate and nitrogen to the kernel could ultimately improve grain yield.

Researchers have demonstrated that ^13^C-photosynthate allocation to grain increased with nitrogen fertilizer (Wei et al., 2019; Ren et al., 2021) but decreased when the nitrogen rate exceeded the acceptable rate (Zhang et al., 2021c). In this study, the nitrogen fertilization improved the ^13^C-photosynthate and nitrogen accumulation and nitrogen distribution ratio to the kernel but reduced the ^13^C-photosynthat distribution ratio to the kernel **Figure 3**).

In addition, ^13^C-photosynthate and nitrogen accumulation and distribution characteristics were influenced by fertilizer type. Compared with common urea, controlled-release fertilizers and BCRF increased dry matter and nitrogen accumulation per plant and promoted its distribution to kernel (Zhao et al., 2010; Vejan et al., 2021). In this study, The NC treatment had a higher LAI and SPAD (not shown in this paper) (higher source) and a similar Fv/Fm value to the NU, resulting in a higher ^13^C-AC and N-AC. The increased DM, ^13^C-AC, N-AC, ^13^C-DR, and N-DR in the BCRF treatments showed that the BCRF facilitated carbohydrate and nitrogen accumulation in plant tissue and subsequent remobilization to the kernels, resulting in an increased kernel per spike, kernel weight, and harvest index (**Figure 8**). These results are consistent with previous research (Qu et al., 2020; Guo et al., 2022). Compared with urea, plants grown with BCRF fertilizers had a similar “sink” (similar KNP and 1000 KW) but a greater “source” (Fv/Fm, ^13^C-AC, and N-AC) and higher “flow” (^13^C-DR, N-DR, and HI), resulting in more carbohydrate and nitrogen accumulation in the kernels and a higher yield (**Graphical abstract**).

### 4.2 Effect of irrigation and nitrogen fertilizer on root length density

Irrigation and nitrogen supply are the two important factors affecting the formation and development of the maize root system (Ning et al., 2015; Chilundo et al., 2017), and an active and deep rooting system was found to be favorably associated with water and nitrogen extraction and grain yield (Aina and Fapohunda, 1986). Root development rates were critical in enhancing plant biomass and cob yield under conditions of deficit irrigation (Flynn et al., 2020). In maize, a mild soil water deficit (50%–60%) resulted in the development of longer lateral roots and an increased root to shoot ratio (Kang et al., 2000), but severe water stress had an adverse effect on lateral root spread (Sampathkumar et al., 2012). In this study, root length density increased from I1 to I2 and decreased from I2 to I3. Compared with I2, the I1 and I3 treatments reduced root length density by 9.0%–14.6% and 10.6%–24.4%, respectively. In other words, both excessive and deficient irrigation amount may inhibit root elongation. In this study, maize plants in deficit irrigation increased root depth (increased root length density) and water extraction from deeper soil profiles (Li et al., 2022) while simultaneously decreasing leaf area to minimize transpiration, resulting in lower water consumption (Pandey et al., 2000).

In the cold–dry season, the effect of irrigation on root density was weaker than fertilizer type, and slow-release fertilization resulted in overall higher root density, above-ground biomass, and grain yield than quick-release fertilization (Chilundo et al., 2017). In this study, nitrogen fertilizer had a significant impact on root length density, increasing from 0 kg N ha^−1^ to 200–240 kg N ha^−1^, but the effect was less than that observed with changes in irrigation. Compared with I3NU treatment, I2NR treatment increased root length density by 26.4% in 0–60-cm soil layer and by 41.0% in 40–60 cm. The result was consistent with other studies (Flynn et al., 2020; Halli et al., 2021). Appropriate water and nitrogen deficiency induced root elongation in search of more water and nutrients. The moderate water-stress and low nitrogen rate treatments resulted in an optimal root distribution defined by increased root length density and a bigger and deeper penetration scale throughout the soil layers, resulting in fewer drought responses and the best WUE and NUE (Wang et al., 2019b). Additionally, the root length density of BCRF fertilizers was greater than that of urea in this investigation, both at the same and reduced nitrogen rates. The blend product consistently supplies sufficient nitrogen to maize crops (Zheng et al., 2020), demonstrating that BCRF can alter the abundance of microbial colonies and improve soil nitrate content, root growth, and nitrogen uptake throughout the maize growing season (Li et al., 2021; Zhang et al., 2021b). Root length density was positive to photosynthesis (Fv/Fm) on the milk stage, harvest index, 13C-photosynthate, and nitrogen distribution ratio. Suitable root length is beneficial to optimize root–shoot ratio and increase dry matter accumulation in aboveground and underground parts simultaneously (Elazab et al., 2016; Ordonez et al., 2020). In this study, the first increased and then decreased with the increase in irrigation level, which was consistent to previous studies (Elazab et al., 2016; Gao et al., 2022). Furthermore, the higher root length density treatment, with higher water and nitrogen assimilating capacity, delayed the leaf senescence process with higher photosynthetic rate (**Figure 4**) and ultimately increased photosynthate and nitrogen accumulation and distribution in kernel (Chilundo et al., 2017). In this study, a higher root length density in I2NR maintains higher maize photosynthesis (Fv/Fm) in the milk stage, delayed leaf senescence in the later stage, and results in similar kernel weights as with I3NU.

### 4.3 Effect of irrigation and nitrogen fertilizer on grain yield, NAUE, and WUE

Water shortage is worsening, and droughts are becoming more common in the Huang-Huai-Hai Plain, China’s key summer maize-producing region (Kang and Zhang, 2016; Chen et al., 2021). Effective irrigation practice is critical for maintaining high summer maize yields while improving WUE. Deficit irrigation is preferable to full irrigation for eco-agriculture (Zhang, 2003a; Tavakkoli and Oweis, 2004; Geerts and Raes, 2009). Researchers have reported that 75% ET_c_ in winter wheat (Lu et al., 2021) and 80% ET_c_ in maize (Guo et al., 2022) produced higher yield and WUE due to higher net photosynthetic efficiency and leaf area index. In this study, the plants in I1 were severely drought stressed and had the lowest Fv/Fm, dry matter, kernel number, kernel weight, and yield but the highest WUE. Fv/Fm, optimal/maximal quantum yield of PSII, was the indicator for adjusting leaf growth status under water deficit (Song et al., 2018; Li et al., 2019). The I1 significantly decreased the maximum light energy absorption and capture efficiency and accumulated low energy for photosynthesis, limiting the maize dry matter accumulation. When compared with I3, the maize growth in I1 was severely limited, with yield losses of 10.9%–32.0%; hence, this treatment is not recommended for maize production. I2 maize had lower dry matter, ^13^C-AC, and N-AC than I3, but it produced the same yield due to higher root length density, HI, ^13^C-DR, and N-DR. This finding was consistent with previous reports (Tolk et al., 1999; Oktem et al., 2003; Payero et al., 2006; Imma and Maria, 2007; Lu et al., 2021; Guo et al., 2022) that appropriate deficit irrigation optimizes yield and WUE. As a result, a mild water deficit of 75% ET_c_ promoted deeper root growth (40–60 cm), maintaining long-term Fv/Fm benefits for leaf photosynthesis, and promoted more photosynthate and nitrogen from other organs to kernel tissues, resulting in increased grain yield and WUE.

The tolerance of maize to drought stress varied depending on the stage of growth. Drought from the tasseling stage to the milk stage had the largest impact on maize output, followed by drought from the seventh leaf stage to the tasseling stage and drought from the sowing to the seventh leaf stage (Zhang et al., 2021a; Zhu et al., 2021). A hypothetical lower degree of drought stress in the sensitive period and a higher degree of drought stress in the non-sensitive period could further improve the yield and WUE than drought stress during the entire growth period. The degree of drought stress based on ET_c_ criteria during the maize growing season needs to be further studied (Mansouri-Far et al., 2010).

Clarifying the relationship between ET and maize grain yield, WUE and NAUE could improve our understanding of regulatory mechanisms when facing persistent water scarcity and climate change (Chen et al., 2021). The trend of grain yield, WUE, and ET in 2018 was consistent to previous studies (Grassini et al., 2009; Hernández et al., 2015). The lower ET for highest grain and WUE in 2016 and 2017 may be attributed to the higher productivity for limited irrigation when rainfall was prevented. Increased with ET, the grain yield were quadratic (**Figure 6**). Without taking soil evaporation into account, larger daily ET rates could be the result of increased root capacity for water and nitrogen extraction (Canales et al., 2021) and/or a greater canopy capacity, resulting in higher photosynthate accumulation (Hernández et al., 2015). While photosynthesis increased initially and then stayed consistent as leaf stomatal opening increased, excessive stomatal opening resulted in excessive water loss and decreased the leaf immediate water use efficiency. Furthermore, the grain yield in 2016 and 2017 under rainproof shelter was significantly lower than that in 2018 and the farmer field in this region. With rainproof shelter, the irrigation effect on maize performance could be studied clearly, reducing the risk of unforeseen rainfall affecting (Kundel et al., 2018). However, the temperature was higher than the field, resulting in higher evapotranspiration, shorter growth period, and lower yield. The maize performance under rainproof shelter could provide referential value for dryland or dry years.

The actual average ET (526.2 mm) for I3 in 2018 was much higher than the average ET_c_ (335.9 mm, calculated by FAO56) from 1981 to 2015, which was higher than the precipitation in 2016 and 2017 but lower than the precipitation in 2018 (349.4, 193.6, and 389.8 mm in the three growing seasons, respectively). The discrepancy between real ET and estimated ET_c_ was partly related to an imbalance in the timing and quantity of rainfall and maize need (Liu et al., 2022b). The ineffective evaporation was compounded by more precipitation prior to the V12 stage (231 mm) but a lower maize demand (94.6 mm). Rainfall exceeds maize demand by a substantial margin, resulting in significant water losses through soil evaporation (Jia et al., 2021) and leachate (Nouchi et al., 1995; Li et al., 2020), whereas the rainfall (15.6 mm) was much lower than the maize demand (61.6 mm) during V12 stage to anthesis stage, the water critical period. The low yield and WUE of I0 treatment throughout the three seasons indicated that additional irrigation was required for the summer maize season, despite the fact that rainfall was greater than ET_c_ in 2018 (Ren et al., 2022).

Improved grain yield and NAUE requires better coordination of crop nitrogen requirements and multiple-source availability (Cui et al., 2010; Ciampitti and Vyn, 2012; Zhang et al., 2012; Meng et al., 2016). A larger LAI and SPAD (not shown) and a similar Fv/Fm value (for the same nitrogen rate treatment) in the NC treatment resulted in increased ^13^C-AC and N-AC. The higher dry matter, ^13^C-AC, N-AC, ^13^C-DR, and N-DR in the BCRF treatments indicate that the BCRF enhanced the carbohydrate and nitrogen accumulation in plant tissue and subsequent remobilization to the kernels, ultimately resulting in greater yield. These results are consistent with previous research (Qu et al., 2020; Guo et al., 2022). Compared with plants grown using urea, plants grown with BCRF fertilizer had the same “sink” (similar kernel number per spike) and higher “source” (^13^C-AC and N-AC) and “flow” (^13^C-DR, N-DR and HI), resulting in more carbohydrate and nitrogen accumulation in the kernels and a higher yield **Graphical abstract**.

The coupling effect of irrigation amount and nitrogen management was significant. Blending control-release fertilizer and urea could dramatically alleviate grain yield loss due to water stress (Guo et al., 2022). In this study, the grain yield of NC was 6.6%–10.6%, 0.9%–3.5%, and 0.9%–2.0% higher than that of NU in the 2016, 2017, and 2018 growing seasons, respectively. However, the difference between NU and NC was greater under I2 and I3 treatments. In addition, drought stress was lessened because of the nitrogen fertilizer (Tilling et al., 2007; Sandhu et al., 2019). In this study, the nitrogen-fertilized treatments, especially the I1 and I0 treatments, significantly increased the Fv/Fm, ^13^C-AC, N-AC, and grain yield compared with the N0 treatments. Drought-stressed maize had a lower root density (Chilundo et al., 2017; Gheysari et al., 2017), excessive nutrients remaining in the soil (Ge et al., 2012), and a reduced nitrogen uptake (Xiao et al., 2021). Proper irrigation (I2 in this study) helped to enhance N-AC and ^13^C-AC in the kernels and NAUE. Excessive irrigation resulted in ineffective plant development and decreased N-DR content in kernels and raised the danger of nutrient leaching (Ren et al., 2016; Srivastava et al., 2020).

## 5 Conclusion

Compared with the I3NU treatment, the I2NC and I2NR treatments increased the kernel number per ear (sink size), maintained a higher Fv/Fm in the milk stage (Ministry of Water Resources), increased ^13^C-photosynthate and nitrogen accumulation, and promoted ^13^C-photosynthate and nitrogen transport from nutritive organs to the kernels (flow), resulting in a higher harvest and a comparable yield. Meanwhile, I2NC and I2NR had a reduced irrigation input and topdressing cost while synchronously increasing the WUE and NAUE. Due to its balanced “source-flow-sink” characteristics, the 75% ET_c_-based irrigation combined with 200 kg N ha^−1^ of BCRF is an effective treatment in terms of yield, WUE, and NAUE. Additional field experiments on a 75% ET_c_ irrigation treatment with different water deficits in the water-sensitive and water-insensitive stages of the plants should be undertaken to optimize the potential for greater yields and resource use efficiency.

## Data availability statement

The original contributions presented in the study are included in the article/**Supplementary Material**. Further inquiries can be directed to the corresponding authors.

## Author contributions

LG: data curation, writing—original draft, visualization, investigation, and funding acquisition. XM: writing—review and editing. JQ: supervision. BT: funding acquisition and supervision. WZ: conceptualization and writing—review and editing. LX: conceptualization and writing—review and editing. All authors contributed to the article and approved the submitted version.
