# Supplementary material for: Nitrogen reduction combined with ETc irrigation maintained summer maize yield and increased water and nitrogen use efficiency
Source: Front Plant Sci. 2023 Jun 22;14:1180734. doi: 10.3389/fpls.2023.1180734 (PMC10324978; doi:10.3389/fpls.2023.1180734)
Supplement: Supplementary file 1 [file Table_1.docx]

Supplementary Material

A combination of ET_c_-based deficit irrigation and reduced N rate of does not decrease grain yield but increases water and nitrogen use efficiency in summer maize

Limin Gu ^1,2^, Xinyuan Mu ^1^, Jianshuang Qi ^1^, Baojun Tang ^1^, Wenchao Zhen ^2,3†^, Laikun Xia ^1†*^

Correspondence:

Wenchao Zhen, wenchao@hebau.edu.cn

Laikun Xia, [xialaikun@126.com](mailto:xialaikun@126.com);

**Supplementary Tables 1** Effect of irrigation, nitrogen fertilizer, and their interaction on ^13^C-photosynthate (or nitrogen) accumulation and distribution ratio during 2016–2018 (F values and statistical significance).

| Year | Treatment | ^13^C-AC | | ^13^C-DR  of kernel | N-AC | | N-DR  of kernel | Fv/Fm  on silking stage | Fv/Fm  on milk stage | 0-20  cm  RLD | 20-40  Cm  RLD | 40-60  cm  RLD | Root-shoot ratio |
| --- | --- | --- | --- | --- | --- | --- | --- | --- | --- | --- | --- | --- | --- |
|  |  | Whole plant | kernel |  | Whole plant | Kernel |  |  |  |  |  |  |  |
| 2016 | F values (Irrigation) | 130.54** | 218.18** | 68.4** | 121.42** | 107.56** | 77.9** | 277.07** | 338.04** |  |  |  |  |
|  | F values (Nitrogen) | 236.11** | 539.74** | 61.9** | 440.85** | 677.55** | 36.2** | 94.95** | 38.87** |  |  |  |  |
|  | F values (Irrigation×Nitrogen) | 5.52** | 4.18** | 8.7** | 3.21** | 2.12** | 11.6** | 2.29* | 0.73ns |  |  |  |  |
| 2017 | F values (Irrigation) | 265.31** | 231.86** | 16.2** | 941.48** | 1255.39** | 7.79** | 415.86** | 356.34** |  |  |  |  |
|  | F values (Nitrogen) | 535.58** | 246.82** | 123.8** | 638.33** | 741.43** | 35.99** | 32.35** | 173.98** |  |  |  |  |
|  | F values (Irrigation×Nitrogen) | 5.72** | 11.36** | 9.8** | 10.43** | 28.72** | 17.56** | 2.39* | 8.26** |  |  |  |  |
| 2018 | F values (Irrigation) | 611.71** | 1069.59** | 103.1** | 408.24 | 468.07** | 8.49** | 383.98** | 320.26** | 165.2** | 120.7** | 93.8** | 29.6** |
|  | F values (Nitrogen) | 568.65** | 1348.65** | 57.5** | 840.63** | 490.76** | 37.98** | 143.38** | 185.73** | 106.4** | 21.9** | 36.3** | 36.2** |
|  | F values (Irrigation×Nitrogen) | 22.48** | 18.2** | 20.7** | 1160.06** | 6.99** | 3.55** | 11.62** | 18.07** | 7.5** | 3.0* | 5.2** | 14.1** |

**Note:** ^13^C-AC, ^13^C-photosynthate accumulation; N-AC, nitrogen accumulation; Fv/Fm, maximum photochemical efficiency of photosystem II; RLD, root length density. Different letters in the same column indicate significant differences at the 0.05 level. *: significant at P ≤ 0.05; **: significant at P ≤ 0.01, NS: not significant at P ≤ 0.05.
